# The feasibility and acceptability of a brief psychological intervention for adults with long-term health conditions and subthreshold depression delivered via community pharmacies: a mixed methods evaluation—the Community Pharmacies Mood Intervention Study (CHEMIST)

**DOI:** 10.1186/s40814-022-00992-7

**Published:** 2022-02-03

**Authors:** Carolyn A. Chew-Graham, Charlotte E. W. Kitchen, Samantha Gascoyne, Elizabeth Littlewood, Elizabeth Coleman, Della Bailey, Suzanne Crosland, Caroline Pearson, Shehzad Ali, Jay Badenhorst, Clare Bambra, Catherine Hewitt, Claire Jones, Ada Keding, Dean McMillan, Claire Sloan, Adam Todd, Paul Toner, Cate Whittlesea, Michelle Watson, Simon Gilbody, David Ekers

**Affiliations:** 1 School of Primary, Community and Social Care, Keele University, Keele, UK; 2 Department of Health Sciences, University of York, York, UK; 3 Department of Epidemiology and Biostatistics, Western University, Ontario, Canada; 4 Whitworth Chemists Ltd, Foxhills Industrial Estate, Scunthorpe, UK; 5 Institute of Population Health Sciences, Newcastle University, Newcastle-upon-Tyne, UK; 6 Public Health Team, Adult & Health Services, Durham County Council, Durham, UK; 7 Hull York Medical School, University of York, York, UK; 8 School of Pharmacy, Newcastle upon Tyne, UK; 9 Centre for Improving Health-Related Quality of Life, School of Psychology, Queen’s University Belfast, Belfast, UK; 10 UCL School of Pharmacy, University College London, London, UK; 11 Research and Development, Tees, Esk and Wear Valleys NHS Foundation Trust, Middlesbrough, UK

**Keywords:** Community pharmacies, Public health, Long-term conditions, Subthreshold depression, Multi-morbidity, Psychological intervention, Mental wellbeing, Behavioural activation, Feasibility study, Mixed methods

## Abstract

**Background:**

Adults with long-term health conditions (LTCs) are more likely to experience depressive symptoms which can worsen health outcomes and quality of life, and increase healthcare costs. Subthreshold depression may go undetected and/or untreated.

The Community Pharmacies Mood Intervention Study (CHEMIST) explored whether community pharmacies represent a suitable setting to offer brief psychological support to people with LTCs and comorbid subthreshold depression.

**Methods:**

A feasibility intervention study with a nested mixed methods evaluation was employed. Adults with subthreshold depression and a minimum of one LTC were recruited from community pharmacies/local general practices and offered a brief psychological support intervention (‘Enhanced Support Intervention’ (ESI)), based on behavioural activation within a Collaborative Care framework. The intervention included up to six sessions supported by pharmacy staff (‘ESI facilitators’) trained to deliver the ESI within the community pharmacy setting.

Recruitment, retention rates and engagement with the ESI were assessed. Semi-structured, one-to-one interviews with pharmacy staff and study participants, and a focus group with pharmacy staff, explored experiences and acceptability of the study and the ESI. Themes were mapped onto constructs of the Theoretical Framework of Acceptability.

**Results:**

Recruitment of ESI participants was challenging and slower than anticipated despite the varied methods of recruitment employed; although, this was useful in identifying barriers and enabling factors for participation. Engagament with the ESI was good with n=17 (71%) recruited participants commencing the ESI. The ESI was found to be acceptable to participants and ESI facilitators. Retention rate at 4 months was good n=20 (87.0%).

The main barriers to identifying potential participants for pharmacy staff were lack of time, resources and limited experience in research. The ESI training and support manual were acceptable to ESI facilitators. The ESI and supporting patient workbook were acceptable to people with LTCs and subthreshold depression.

**Conclusions:**

Community pharmacies were viewed as an acceptable setting in which to deliver preventative brief psychological support to people with LTCs at risk of depression.

This feasibility study provided important data to inform the design of a pilot randomised controlled trial in this setting and highlighted important considerations for future pharmacy-based research.

**Trial registration:**

ISRCTN11290592

## Key messages regarding feasibility

- Prior to this feasibility study, it was not known whether community pharmacies are a suitable setting to offer brief psychological support to people with LTCs and comorbid subthreshold depression, and whether such an intervention would be acceptable to pharmacy staff and this patient group. The barriers and facilitators to recruitment were unknown.
- The research team considers that the community pharmacy could be an ideal setting to deliver a psychosocial intervention to older adults, particularly in deprived areas, with subthreshold depression, reducing the stigma associated with low mood in older adults. Our feasibility intervention study with a nested  mixed methods evaluation showed that the intervention was acceptable to our study participants. We demonstrated however that there were several barriers to recruitment which would need to be overcome in a full trial and in future implementation of this intervention.
- Study processes need streamlining to facilitate embedding the study within the community pharmacy setting. A variety of recruitment strategies are also required with a need to extend beyond pharmacy-based recruitment approaches, and should include a wider collaboration with general practitioners (to undertake searches of practice lists) and the wider community and support services.

## Background

Up to 30% of the UK population have long-term physical health conditions (e.g. heart disease, diabetes, arthritis), which accounts for 70% of the National Health Service (NHS) expenditure [1]. Depression is two to three times more prevalent in people with long-term conditions (LTCs), compared with the general population, and accounts for 4.3% of the global disease burden, causing 63 million disability adjusted life years annually [2, 3]. Depression alongside LTCs can worsen health outcomes and quality of life, reduce the ability to self-manage and double healthcare costs [2]. Subthreshold depression, defined as depressive symptoms which fall below criteria for a diagnosis of major depression [4], is highly prevalent, impacts on functioning and quality of life, and is a major risk factor for progression to major depression [5]. Subthreshold depression can be identified by a positive screen on the Whooley depression questions [6], but not meeting the threshold for a depressive illness. People with comorbid subthreshold depression and LTCs are more likely to live in more deprived areas, contributing significantly to health inequalities [7].

For many people with subthreshold depression alongside long-term physical health conditions, symptoms may go undetected and untreated. Primary care and mental health services struggle to meet the demands of depression, with over 80% of ‘below threshold’ conditions remaining untreated [8]. Recent research suggests that psychological interventions can reduce depressive symptoms in people with subthreshold depression and reduce the incidence of major depression [9], but they are not commonly available. To overcome this challenge, there is a need to place less focus on traditional health service providers and more emphasis on viewing subthreshold depression as a public health priority requiring new management approaches.

Community pharmacies have been identified as an easily accessible and cost-effective platform for delivering health care and public health services worldwide [10]. They are an integral part of NHS primary care services and offer people a link to local health and social care [11, 12]. Community pharmacies already play an active role in health promotion and are well placed to offer opportunistic support to people with a range of health problems, including subthreshold depression. Moreover, people with LTCs have regular contact with their pharmacy, which might provide an opportunity to reach out to people experiencing subthreshold depression who may otherwise not have access to support. Community pharmacies also have a strong presence in poorer communities, and potentially offer a less stigmatising place to identify and offer support for mood problems [13, 14]. Given that 89% of people live within a 20-min walk of a community pharmacy, this may represent a convenient and plausible public health setting to offer people brief psychological interventions [14].

A large UK-based randomised controlled trial (RCT) conducted with people over 65 years (the CASPER trial) with subthreshold depression (most with at least one LTC), found that a collaborative care (CC) intervention was effective and acceptable to older adults [15]. The CC intervention included behavioural activation (BA), which focuses on activity scheduling to encourage people to resume activities that they may have previously enjoyed but are currently avoiding, or develop new activities that take into account life changes (such as physical health problems or bereavement). In the CASPER trial, the intervention was supported by structured phone or face-to-face sessions delivered by a non-medical specialist (a case manager), regular use of a mood measurement questionnaire, and liaison with the General Practitioner (GP) involved in the care of the patient, if needed [15]. The CASPER intervention was shown to reduce depressive symptoms at the 4th- and 12th-month follow-ups, and nearly halved progression to major depression, compared with usual primary care [15].

BA delivered by Practice Nurses, who already provide ongoing monitoring and support to people with LTCs, is a promising approach [16, 17]. There is emerging evidence of the feasibility of delivering BA by nonspecialist mental health professionals [18, 19], and practitioners outside the NHS [20]. The content of a BA intervention for subthreshold depression shares much with other public health interventions, such as smoking cessation or weight management (e.g. goal setting, facilitated self-help and diary keeping) already delivered as part of the Healthy Living Pharmacy programme in community pharmacies [21]. Therefore, a BA intervention, targeted at people with subthreshold depression, may also lend itself for delivery by pharmacy staff trained to implement public health behavioural change programmes.

The Community pHarmaciEs Mood Intervention STudy (CHEMIST) adapted an existing BA intervention [15] for people with subthreshold depression delivered within a CC framework [16]. It aimed to determine whether this psychological intervention (termed ‘Enhanced Support Intervention’ (ESI)) could be delivered by suitably trained community pharmacy staff (ESI facilitators) to adults with subthreshold depression and LTCs. We report a mixed methods evaluation of the feasibility of conducting CHEMIST. The quantitative evaluation reports the feasibility of recruitment, engagement with the ESI and retention rate. The qualitative evaluation explored the experiences of the ESI with ESI facilitators and participants offered the ESI within the community pharmacy setting. This feasibility study enabled refinement of the ESI and study procedures in advance of a pilot RCT [22].

## Methods

### Design

A feasibility intervention study with a nested mixed methods evaluation.

### Population

Adults (aged 18 years or over) with a minimum of one LTC (arthritis, cancer, cardiovascular conditions, diabetes, respiratory conditions, stroke, progressive conditions such as Parkinson’s disease) and comorbid current subthreshold depression (determined by two to four symptoms of depression (score of 2–4) on the major depressive module of the Mini International Neuropsychiatric Interview (MINI) [23]). People who were drug or alcohol dependant, or with active suicidal ideation, a cognitive impairment, bipolar disorder/psychosis/psychotic symptoms or who were currently receiving psychological therapy were excluded.

### Intervention

All eligible participants were offered the ESI, and no care was withheld. The ESI was a modified form of a CC/BA intervention for subthreshold depression validated in previous studies in UK primary care [16]. Community pharmacy staff with roles such as pharmacy managers, dispensers, healthy living advisors and counter assistants with experience in health promotion programmes were recruited and trained to deliver the ESI. The training involved attending a two-day ESI facilitator workshop—workshops included community pharmacy staff from across recruiting pharmacies and were held mainly off-site. The workshop trainers were experienced in BA/CC and had a variety of backgrounds including clinical psychology, psychological services and health care services. Following the training, ESI facilitators were required to complete and pass a telephone-based competency assessment before delivering the ESI. The ESI was supported by an ESI facilitator manual.

The ESI included up to six sessions, delivered either face-to-face or over the telephone, over a maximum 4-month period. The first session was intended to last up to an hour with subsequent sessions lasting up to 30 min. The ESI involved BA using facilitated self-help, goal-orientated activity scheduling, with a focus on identifying aspects of the participant’s life that were having a detrimental impact on psychologically healthy activities, and scheduling activities to become and stay well. A patient workbook was provided and intended to be used during and between sessions.

Recruited participants (‘ESI participants’) were proactively followed up by their ESI facilitator who monitored their depressive symptoms at each session and signposted them to other services (for example their GP or local community resources) where necessary. ESI facilitators were supervised on a session-by-session basis by a clinical supervisor, who was a member of the research team. In the supervision sessions, any questions that arose relating to the intervention were discussed. The supervisor worked collaboratively with the ESI facilitator to problem solve any difficulties and share learning from the intervention both within and across similar trials. The supervisor checked that the intervention was being adhered to and that, within session measures and risk monitoring had been conducted at every session. ESI facilitators could contact the supervisor between scheduled supervision sessions, if they needed to.

We considered that an ESI participant had ‘completed’ the intervention if they had participated in at least two sessions, and this was deemed appropriate for the participant circumstances through discussion with their ESI facilitator and the clinical supervisor. We considered an ESI participant to have ‘dropped out’ if they completed only one session.

### Recruitment

#### Recruitment of participants to the feasibility study

Potential participants were identified from community pharmacies in the northeast of England. Community pharmacies were identified and recruited via discussions with our pharmacy stakeholders (including pharmacy co-applicants) and via local pharmacy/research networks. The aim was to recruit between 20 and 30 participants using a variety of recruitment methods, which were refined and/or implemented as the feasibility study progressed based on ongoing feedback from recruiting community pharmacies.

##### Pharmacy-based recruitment

- *In-person approach by pharmacy staff*: Eligible people visiting the pharmacy were invited to receive a study information pack to take away and read.
- *Home delivery*: Eligible customers who receive their prescriptions via home delivery services were provided with a study information pack with their prescription.
- *Pharmacy system search*: Pharmacies identified eligible customers via the pharmacy patient medication record systems, and they were posted a study information pack.
- *Posters*: Study posters were displayed in recruiting community pharmacies, and customers were encouraged to contact the study team directly or speak to the pharmacy staff for more information.

##### GP-based recruitment

- *GP database search*: Research-active GP practices located near to participating community pharmacies conducted searches to identify eligible patients. Study information packs were then posted out via the GP practice.

The study information pack included an invitation letter, participant information sheet, consent form, background information sheet and a prepaid return envelope. Interested customers/patients were asked to return the consent form and background information sheet to the research team. On receipt of a completed consent form, the participant was contacted by a member of the research team and a telephone diagnostic interview to determine study eligibility was conducted.

#### Recruitment of participants to the qualitative study

Participants were asked for consent to participate in a qualitative interview as part of the original study consent. ESI participants were approached for an interview following completion of the ESI. Those participants who did not commence the ESI were also approached to take part. ESI participant interviews were conducted at their home, at their community pharmacy or over the telephone (dependent upon ESI participant preference).

ESI facilitators who completed the ESI training workshop received a study information pack in the post inviting them to take part in the qualitative study. This included an invitation letter, participant information sheet, consent form and a prepaid return envelope. Interested ESI facilitators were asked to complete and return the consent form directly to the research team. This process was conducted independent of the pharmacy to ensure study invitation/involvement was free from coercion from employers and/or co-workers. Those ESI facilitators who returned a consent form were invited to an initial interview to explore their experiences and views of the training immediately following the ESI workshop. These ESI facilitators were also invited to participate in a second later interview to explore their experiences of delivering the ESI once they had delivered this to a minimum of one ESI participant. Other ESI facilitators were interviewed after they had delivered the intervention to a minimum of one ESI participant. Interviews with ESI facilitators were conducted in a private room at the community pharmacy or over the telephone (dependent upon their preference).

In-depth semi-structured interviews were conducted with ESI participants and ESI facilitators. Existing literature was used to develop interview topic guides, which were amended following each interview and as analysis progressed.

A focus group was also held with community pharmacy staff to explore their experiences of the study including recruitment, delivery of the ESI, and the study procedures. All pharmacy staff (including ESI facilitators but also the broader staff responsible for recruitment) within each recruiting pharmacy received a study information pack (including an invitation letter, participant information sheet, consent form and prepaid envelope). Those pharmacy staff interested in attending a focus group were asked to return the consent form to the study team.

### Data analysis

#### Quantitative analysis

ESI participants provided basic demographic information at the point of consent. Eligible participants completed questionnaires at baseline and 4 months post-baseline. These included the PHQ-9 [24] (Patient Health Questionnaire-9 for depressive symptoms), PHQ-15 [25] (Patient Health Questionnaire-15 for somatic symptoms), GAD-7 [26] (Generalized Anxiety Disorder-7 for anxiety symptoms) and SF-12 [27] (Short Form Survery-12 for quality of life). Quality and completeness of these questionnaires were explored to determine their acceptability as outcome measures. As this was a feasibility study, no formal statistical analyses were undertaken, and results are thus presented descriptively.

#### Qualitative analysis

The interviews and focus group were recorded using an encrypted digital audio recorder and were professionally transcribed verbatim. All data were analysed thematically initially using constant comparison [28] followed by a framework analysis [29] using the Theoretical Framework of Acceptability (TFA) [30] to guide the analysis. A team of researchers from different professional backgrounds conducted the analysis, to increase the trustworthiness of the analysis [31] with regular consensus meetings.

## Results

### Quantitative findings

We recruited eight community pharmacies in both rural and urban settings in the north east of England.

**Fig. 1:**
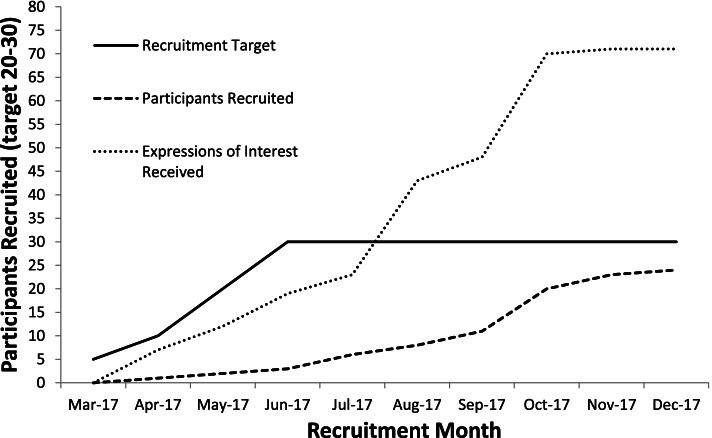
Recruitment activity by month

**Fig. 2:**
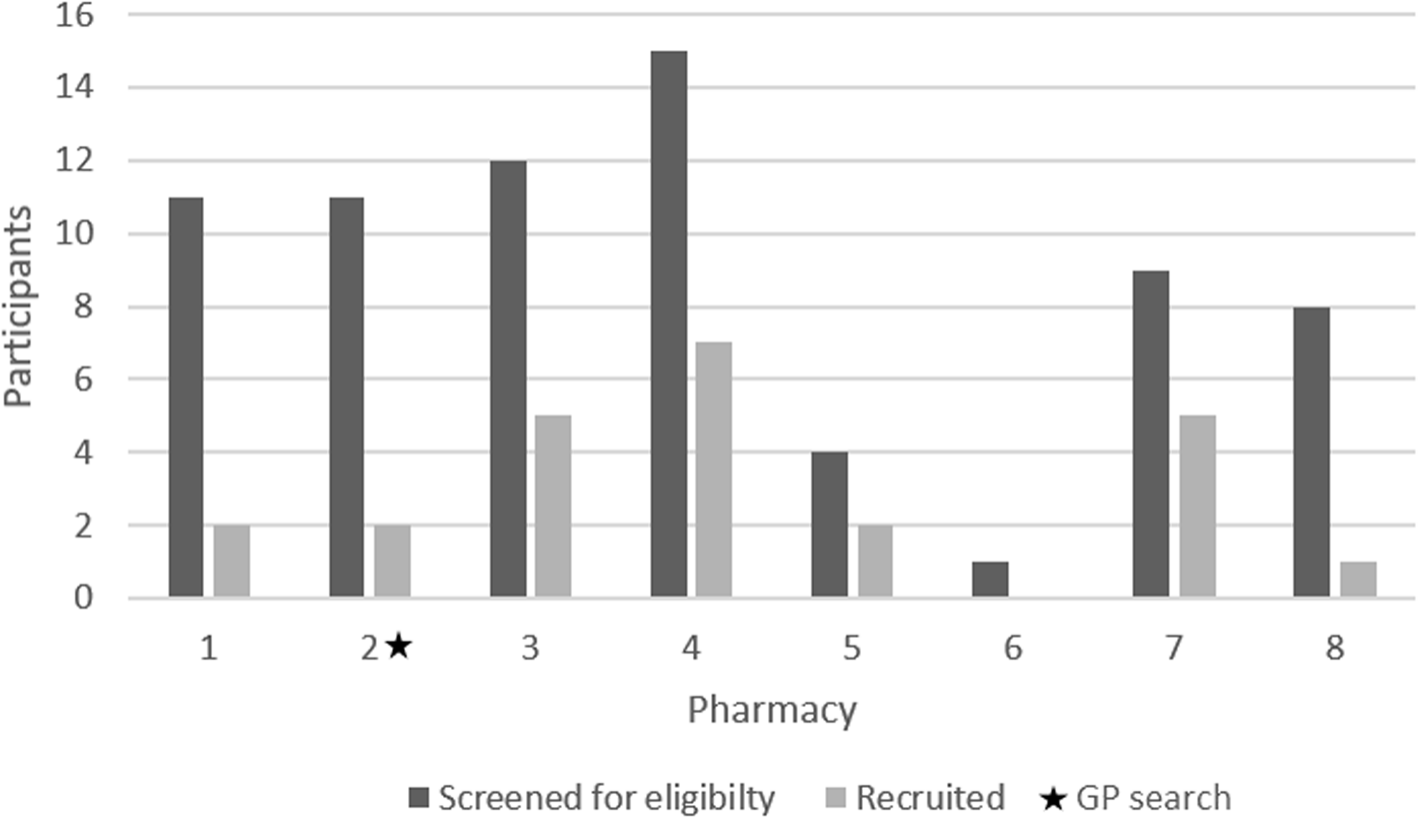
Number of people screened and recruited per pharmacy

A total of 24 ESI participants were recruited over a 9-month period between April and December 2017 (see Fig. 1). Overall, 882 study information packs were distributed across the eight community pharmacies (168 face-to-face in pharmacy, 414 via home deliveries, 300 via one pharmacy system search) and 200 information packs were posted following one general practice database search. A total of 71 people consented to be screened (6.6%), and 28 were eligible to participate in the study (39.4% of those who consented) with 24 (85.7% of those who were eligible) agreeing to take part. The length of recruitment activity across the eight community pharmacies varied from 2.7 to 7.2 months, with an average length of 5.5 months. This gives an average recruitment rate of 0.55 participants per community pharmacy per month. Figure 2 details the screening and recruitment for each community pharmacy.

**Fig. 3:**
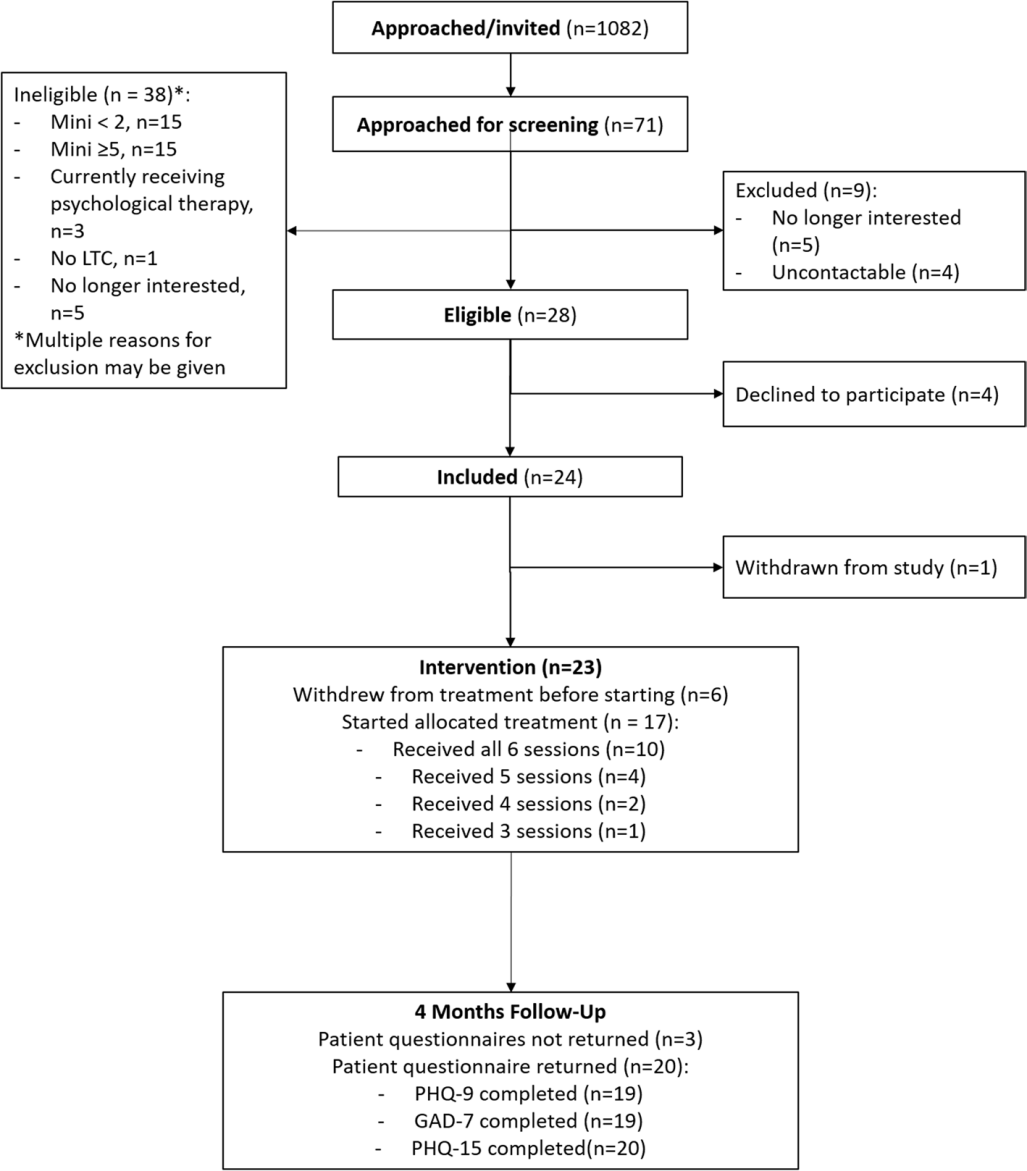
Flow of participants through the study

The most common reasons for study ineligibility were scoring ≥ 5 on the MINI indicating a major depressive episode (*n* = 15), or scoring < 2 on the MINI indicating no current depression (*n* = 15). Further details of reasons for ineligibility can be found in Fig. 3, which also shows the flow of participants through the feasibility study.

### Participant characteristics

Baseline data for the 24 recruited ESI participants are detailed in Table 1. There were no missing data with respect to participant characteristics. The average participant age was 66.8 years, ranging from 51.3 to 83.6 years. All participants classified themselves as of white ethnicity. The majority of participants (58.3%) had not continued with education after the minimum school leaving age; however, six participants had a degree or equivalent level qualification. A variety of health problems were self-reported, with the most common being hypertension (*n* = 16).

**Table 1:** Baseline characteristics

**Age, years**	
Mean (SD)	66.8 (9.8)
Median (min, max)	65.9 (51.3, 83.6)
**Gender;** ***N*** **(%)**	
Male	6 (25.0)
Female	18 (75.0)
**Smoking Status:** ***N*** **(%)**	
Non-smoker	12 (50.0)
Current smoker	4 (16.7)
Ex-smoker	8 (33.3)
**On average, do you drink 3 or more units of alcohol each day?** ***N*** **(%)**	
Yes	1 (4.2)
No	23 (95.8)
Do not know	0 (0.0)
**Health problems:** ^**a**^***N*** **(%)**	
Diabetes	7 (29.2)
Osteoporosis	2 (8.3)
High blood pressure	16 (66.7)
Rheumatoid arthritis	3 (12.5)
Osteoarthritis	9 (37.5)
Stroke	5 (20.8)
Cancer	2 (8.3)
Respiratory conditions	7 (29.2)
Eye conditions	3 (12.5)
Heart disease	8 (33.3)
Other	14 (58.3)
**Did your education continue after the minimum school leaving age?** ***N*** **(%)**	
Yes	10 (41.7)
No	14 (58.3)
**Do you have a degree or equivalent professional qualification?** ***N*** **(%)**	
Yes	6 (25.0)
No	18 (75.0)
**Ethnicity:** ***N*** **(%)**	
White	24 (100.0)
Asian or Asian British	0 (0.0)
Black or Black British	0 (0.0)
Other ethnic group	0 (0.0)
**Number of children:** ***N*** **(%)**	
0	4 (16.7)
1	7 (29.2)
2	8 (33.3)
3	5 (20.8)
4+	0 (0.0)
**Marital status:** ***N*** **(%)**	
Single	1 (4.2)
Divorced/separated	1 (4.2)
Widowed	5 (20.8)
Cohabiting	2 (8.3)
Civil partnership	3 (12.5)
Married	12 (50.0)

^a^Multiple answers could be selected

### Follow-up

A total of 20 ESI participants completed and returned a 4-month follow-up questionnaire (87.0%); this included five participants who did not commence the ESI sessions. One participant withdrew from the study.

### Standardised measures

ESI participant scores on the PHQ-9, GAD-7, PHQ-15 and SF-12 (physical and mental components) are reported in Table 2, both at baseline and 4-month follow-up.

**Table 2:** PHQ-9, GAD-7, PHQ-15 and SF-12 scores at baseline and 4-month follow-up

	Baseline (*N* = 24)	Month 4 follow-up (*N* = 20)
**PHQ-9 Depression (range 0–27)**^**a**^	*N* = 24	*N* = 19^c^
Mean (SD)	13.5 (5.8)	9.9 (6.1)
Median (min, max)	13 (2, 24)	9 (3, 20)
No depression	1 (4.2)	5 (26.3)
Mild depression	4 (16.7)	7 (36.8)
Moderate depression	12 (50.0)	1 (5.3)
Moderately severe depression	2 (8.3)	5 (26.3)
Severe depression	5 (20.8)	1 (5.3)
**GAD-7 Anxiety (range 0–21)**^**a**^	*N* = 24	*N* = 19^c^
Mean (SD)	10.3 (5.0)	6.5 (4.6)
Median (min, max)	10 (1, 21)	6 (0, 16)
No anxiety	2 (8.3)	7 (36.8)
Mild anxiety	9 (37.5)	8 (42.1)
Moderate anxiety	8 (33.3)	2 (10.5)
Severe anxiety	5 (20.8)	2 (10.5)
**PHQ-15 Depression (range 0–30)**^**a**^	*N* = 24	*N* = 20
Mean (SD)	14.8 (4.7)	13.2 (4.5)
Median (min, max)	15 (4, 25)	14 (5, 23)
Minimal depression	1 (4.2)	0 (0.0)
Low depression	2 (8.3)	4 (20.0)
Medium depression	8 (33.3)	7 (35.0)
High depression	13 (54.2)	9 (45.0)
**SF-12 Physical Component (range 0–100)**^**b**^	*N* = 24	*N* = 20
Mean (SD)	30.9 (8.4)	30.8 (8.4)
Median (min, max)	30.1 (16.3, 49.6)	32.0 (17.2, 46.4)
**SF-12 Mental Component (range 0–100)**^**b**^	*N* = 24	*N* = 20
Mean (SD)	35.7 (9.4)	41.1 (10.7)
Median (min, max)	35.7 (19.6, 52.5)	40.9 (17.5, 60.4)

^a^Higher scores are worse, ^b^higher scores are better, ^c^not all responses were able to be scored

The level of completion for these measures was excellent; 100% at baseline, and between 95 and 100% at 4-month follow-up. Although the sample is small, and, as such, no formal comparisons can be made; there does appear to be an observable trend that scores on outcome measures reduce between baseline and 4-month follow-up.

### Intervention delivery

Seventeen of the 24 ESI participants (70.8%) commenced the ESI. Engagement with the ESI sessions is detailed in Table 3.

**Table 3:** Number of sessions attended by participants

	Number of participants
Total number of intervention session completed	
6	10
5	4
4	2
3	1
2	0
1	0
Withdrew from intervention before start of intervention	7

All 17 participants who commenced the ESI completed a minimum of two sessions. Ten of the seventeen completed all six sessions (58.8%). A total of 91 sessions were conducted, of a possible 102, giving an average attendance of 86.1% for those who started the ESI.

### Qualitative findings

Eleven ESI participants were interviewed, from across five of the eight participating community pharmacies (Table 4). Nine ESI participants had completed between four and six ESI sessions (seven participants had completed all six ESI sessions) and two participants did not start the ESI sessions. Interviews with ESI participants explored their views on being recruited into the study, study processes and their experiences of receiving the ESI.

**Table 4:** Participant demographics for qualitative interviews

**Age, years**	
Mean	64.3 (7.7)
Median (min, max)	62.9 (53.1, 79.8)
**Gender:** ***N*** **(%)**	
Male	7 (63.6)
Female	4 (36.3)
**Health problems:** ***N*** **(%)**	
Diabetes	3 (27.2)
High blood pressure	8 (72.7)
Rheumatoid arthritis	2 (18.1)
Osteoarthritis	3 (27.2)
Stroke	2 (18.1)
Cancer	1 (0.9)
Respiratory conditions	4 (29.2)
Eye conditions	1 (0.9)
Heart disease	3 (27.2)
Other	5 (45.5)

Thirteen interviews were conducted with nine ESI facilitators from across seven of the recruiting community pharmacies (Table 5). Four ESI facilitators completed two interviews (the first on their experiences following the ESI training workshop, the second focused on their experience of delivering the ESI). Five ESI facilitators completed a single interview on their experiences of ESI training and delivery.

**Table 5:** ESI facilitator demographics

**Gender:** ***N*** **(%)**	
Male	1 (11.1)
Female	8 (88.9)
**Job role:** ***N*** **(%)**	
Accuracy checking technician	1 (11.1)
Counter assistant	2 (22.2)
Dispenser	5 (55.5)
Trainee dispenser	1 (11.1)

Five pharmacy staff participated in the focus group.

We report the following themes following application of the TFA [30]: Intervention Coherence, Perceived Effectiveness, Self-efficacy, Burden, Opportunity Costs and Affective Attitudes. Data relating to the construct of ‘Ethicality’ were not present in the transcripts.

Data are presented to support analysis and labelled by identifier and number: *P*, ESI participant; *ESI*, ESI facilitator; *Pharm*, pharmacy staff (focus group).

#### Intervention coherence

This construct assesses the extent to which the ESI participants understand the ESI and how it works.

The identification of mood problems and the provision of subsequent support made sense to ESI participants who received (‘patients’) and delivered (‘ESI facilitators’) the ESI. All participants acknowledged the link between physical and mental health and ill health:

*Well it's gotta be [linked] cos if you're getting up every day feeling poorly it's, you're not gonna be feeling happy, are you, really? So obviously if you get up, you're not well, you stop doing things, makes yer pretty miserable, doesn't it?* (ESI2)

*I do focus on the physical disabilities now as causing me great unhappiness and depression because I can't see any improvement, I, I, and I couldn't look to any improvement, I just felt as if my life was over*. (P049)

The actual ESI seemed to make sense, with ESI participants reflecting on the usefulness of the ESI patient workbook, the structured nature of the ESI, and the ‘homework’ required of them:

*Yes, it did. It was particularly in the workbook, it mentioned things like can't be bothered, not going out, making excuses; and I thought yes, that, that, that's me, but I thought it was just getting older and, you know, not wanting to do things like that, so. That wa, that was, that was very useful.* (P018)

*I thought, and I, I, perfectly honest, it has done me the world of good. It's been therapeutic, because I was getting a regular call on a Wednesday, and I would work to that call on the Wednesday, because I was given a plan, I was given advice, keeping me diary, for example, on how I felt from day to day, planning to do tasks, even though they seemed insurmountable at times…* (P049)

The community pharmacy was seen as an appropriate setting within which to deliver the ESI, with the location seen as familiar and non-stigmatising:

*We have a lotta people that come in and tend to, for whatever reason, see it, us in a pharmacy, as someone as a, who they can talk to openly and honestly.* (ESI1)

ESI participants also reflected on the importance of confidentiality, which appeared to give them confidence to engage in the ESI, that it could produce a positive outcome or ‘*good result’*:

*I know [the facilitator], I can speak to [her] and I know it'll be confidential and it wouldn't go any, any further, it wouldn't go any further with any of the staff or anything like that, she would give me a good result and I would give her a good result…* (P022)

#### Perceived effectiveness

The TFA construct of ‘Perceived Effectiveness’ (PE) describes the extent to which participants perceive that the intervention will achieve its purpose [30]. A number of aspects of the ESI were perceived as likely to achieve their purpose, although some ESI participants disclosed initial uncertainty about the initial assessment:

*Yeah, I thought it was a bit (sighs) a bit strange at first, and then when you read and understand what everybody's trying to say and get to and get your answers from you, I think it, it's done in a very sensitive way and I think it, it's positive.* (P014)

The materials were thought to achieve their purpose, in particular, the patient workbook was well received by ESI participants and also by ESI facilitators who described working through them with the ESI participants:

*We went through it together. So she was like asking us the questions out the book that I already had, you know, so went through together. So yes, it was all right, I understood it, yeah.* (P006)

*I thought that was fine, I just, I thought it made more sense once I got the self-help work book, everything, once I got that, I read this from beginning to end. So I didn't even, you know, I, once I got this I thought oh so we're gonna be doing this and gonna be doing that; but that's the person I am. Some people might just do one stage as, at a time, I read the whole thing to see what it was gonna entail before; so I was well aware what I was going into, and if there was something I didn't like I would have said; but I thought oh this is all right, seems all right to me. So, there you go.* (P002)

Although some ESI participants described how they did not continue with writing in the workbook.

*I gave up filling the end part of the book up cos I thought what activities do yer like to keep, help yer keep well? There's a lotta things that I could, I could be doing more, but that's gonna come in time. I'm more worried about, I'm, was more interested in filling this chart* [activity planner] *to say, right, this is what I did.* (P002)

#### Self-efficacy

Self-efficacy refers to ‘the participant’s confidence that they can perform the behaviour(s) required to participate in the intervention’ [30]. ESI facilitators expressed mixed views about whether they had developed the confidence to deliver the ESI following the ESI training. They particularly expressed concerns about anxiety and uncertainty around assessment of risk:

*…OK. I think one of the questions I would, I would find it quite awkward asking people where you had to just say have you thought about killing yerself in the last week; I think that might come across as, I don't know what the word is, a bit blunt. (laughs) But yeah, apart from that…* (ESI6)

Though ESI facilitators described how they gained confidence with increasing experience of intervention delivery:

*I didn't really find it difficult. I found, I did, at first, at first, when I first started, I thought asking the questions, the risk questions I'd ask'd be quite daunting and impersonal and whether they would be quite negative, but when we've done it on a regular basis I can see that there's a need for it and you can see, especially with one, with one of them, even with the one that decided at four weeks decided she didn't want to go any further, it was a noticeable difference in the scores that it had improved, so at least there was some benefit. So with regards to that, I think it was quite OK, to be honest, I didn't really have any problems with that.* (ESI1 2nd interview)

ESI participants all described confidence in goal-setting, keeping diaries and being monitored by their ESI facilitator:

…*in particular when we were talking about breaking activities down into smaller parts, because again me mobility has really restricted me with a lot of things, and even a simple thing like making the bed (laughs) you, you know, we used that as an ex, one, one example, and it was a case of not making it and changing the blankets all in one go and, you know, so we did it sort of like, you know, with [*names ESI facilitator*] help we, we broke that down into smaller steps.* (P046)

Not all ESI participants were confident that they could continue to practice what they had learned during their work with the ESI facilitators:

*I think I try to now, yeah, I think on things a bit more. Whereas before I would just sit, now I try to get up and do something and I try to have a different outlook on, on the way it was to the way it is now, even though I've got the pressure of my wife it's still, it's still a bit different than what it was.* (P014)

Other ESI participants, however, described continued use of BA techniques learned, including monitoring mood:

*Having a mood chart but; I mean I've, I'm using me own calendar and I'm putting me score; so I just do, I just do morning and night, because morning I'm usually, nine times out of ten, I'm very high in a morning, because I like getting up, I'll get up early, I do everything on a morning, so my mood's quite high, it's when I get, after work I'm tired. Obviously, it lowers and things like that, and at the minute it's, it's on about a 5 when I come back from work, cos when I come back from work I'm thinking about me sister, so. But it'll, it'll be like that, and I'm not worried about that being low when it's been an 8 on the morning, because you haven't had time to think, you know, things like that…* (P002)

#### Burden

Burden refers to ‘the perceived amount of effort that is required to participate in the intervention’ [30]. Both ESI facilitators and ESI participants alluded to the burden that participating in the study entailed. For example, ESI facilitators described the challenge of discussing the study with pharmacy customers, and specifically the difficulty of finding the words to describe the purpose of the study and the description of ‘mood’:

*Like my big problem was how to initially say to people do you want to participate, cos I didn't want to say it's about low mood or sub-threshold depression, I just didn't wanna mention those terms cos I just knew people would be put off. So I was like I need some key words to throw in here (laughs) and she said "Say it's a psychological wellbeing study". And as soon as she said that I was like, right, that's much easier (laughs) I approached a lot more people then, yeah.* (ESI4)

Additionally, pharmacy staff also discussed the burden of the recruitment process:

*...cos we, we've over fourteen thousand items every month, so we have a high patient turnover, so a lot of the staff would only know a handful of patients each. So we're not in a position to sit and, or know that that patient might be eligible or things like that. So I think it would be suitable in the future, but at this stage of the trial, where they're still testing the things out, it doesn't work particularly well for that area, but, and I think that's more to do with the socioeconomic of where that pharmacy's based, rather than anything else.* (FG P3)

All ESI facilitators and pharmacy staff described some difficulty fitting aspects of the study into their daily routine:

*it's sort of fifteen/twenty minutes taken off my time and I'm very needed on the shop floor. So, it means that somebody has to drop down, and then I feel like I've left two very capable dispensers having to cover a shop floor with a queue full of people, which sometimes I do feel guilty about.* (ESI1 2nd interview)

However, this ESI facilitator added:

*But then when you think of the impact it's gonna have on the customers that we see, it's worthwhile doing; regardless of how many staff we've got on the counter, if we can help just one person it's been worthwhile.* (ESI1 2nd interview)

And the ESI facilitator was keen to stress the generalisability of the training and skills learned in being part of the study:

*Yeah, and it's expanded my knowledge, it's made me aware to, to look for what's in our community, not further afield, because people don't want to travel, but to see what's openly available. So now I've got a list of what's openly available for people who are overweight or struggling with anything and; men's football clubs for the over fifties, running clubs. I've been looking at everything so that if anybody says to me I need to do that, I've got a folder; and I would never have done that if it hadn't have done the CHEMIST study.* (ESI1 2nd interview)

Participants in the focus group shared their concerns over the burden and impact of participating in the study:

*A bit, like you said, like it's the, the, [Facilitator] would have to go, she'd want to sit and prepare before somebody came in, to read the notes, to see where she was at. I think the first person that came in took about three-quarters of an hour and then, then there was the phone call with* [names clinical supervisor] *to then catch up; it just seemed to take a lotta time and I know, obviously I, there was another three pharmacies in my group, one of them didn't do it because they do like seventeen thousand, like eighteen thousand items, just don't have time to do it, they said they just don't have time to get the staff to go and give the packs out, never mind seeing people; and it's, it's unfortunately about money...* (FG P5)

Descriptions of burden from ESI participants, however, were seen infrequently; rather, the sessions with the ESI facilitators were seen positively:

*In fact when she said when it was the last one I was quite disappointed really because it was becoming a regular on the Wednesday morning, you know, about an hour we would have talking together.* (P049)

A minority of ESI participants described lack of motivation impacting on their ability to complete the ESI:

*...but it was just the, just the, just the mood I was in, I would say to [the Facilitator] "I'll come in tomorrow". And then when the, the tomorrow come, I thought oh I, I really didn't want to come, you know. but I did, I mean I did do it all, I finished it; took a bit of a while. But just, like I said, all depending; I mean today, the way my hand is, I, I found difficulty driving, to tell you the truth, cos I had to drive with them two fingers. So there is times where I've got to push meself, otherwise I'm just in the house, you know. But, but yeah, I, I, I, I think it's, like I say, I still come, but it was a bit difficult sometimes not wanting to come, so.* [P006]

#### Opportunity costs

The TFA domain of ‘Opportunity Costs’ describes if benefits, profits or values need to be relinquished in order to engage in the intervention [30]. Discussion of opportunity costs was reported by ESI facilitators and pharmacy staff in terms of the time cost on regular pharmacy work due to the ESI facilitator delivering the ESI:

*So I think it's got a big impact on the pharmacy, if you're working in a busy pharmacy, cos you're taking somebody away for a good thirty minutes and then, which is a good hour, because then you have to call* (supervisor) *and then you go through the session with (*supervisor*); so you're talking about, sometimes that can be another fifteen/twenty minutes, depends. So you're taking like a, a body out of the pharmacy; so while that, while that's getting done my work at the back's not getting done.* (FG P1)

ESI participants did not feel that being involved in the study had any impact on their day-to-day activities (‘opportunity costs’), but one ESI participant acknowledged the difficulty of fully participating in the study due to a recent bereavement:

*But I can only do; and obviously with just losing me sister, everything's gone a bit pear-shaped at the minute, but I can only do so much and it, it, it is what it is, but I will get there.* (P002)

#### Affective attitudes

Affective Attitudes describes participants’ feelings about the intervention [30]. That the ESI facilitators were known to ESI participants appeared to be a key enabler of their positive feelings towards participating in the study and working with the ESI facilitator to complete the ESI:

*…but knowing the pharmacy, this particular pharmacy as I do, I wasn't surprised that they were interested in it.* (P018)

…*I think a big part of it is like how the staff are with the patients, like you said you know your patients very well...* (FG P3)

Additionally, the personal qualities of the ESI facilitators were deemed to be important in relationship-building and remaining with the ESI:

*Oh great, absolutely great, great, because she's [the Facilitator] friendly, she's approachable, you know what I mean, so; and I mean that, that's what you need because it, it, some people can find it intimidating*. (P002)

*No, no, I just, I really found* [names ESI facilitator] *to be very helpful, very understanding; there was one time, again it was me mobility, I couldn't get to the chemist, she was quite happy to change the appointment, you know, and, and she was just really, really helpful.* (P046)

The training was valued, not just to help in the delivery of the study, but to assist in routine work within the pharmacy:

*I found that [training] really interesting, and I thought my role as the healthcare assistant, the counter assistant, I could put, put to good use, and obviously with seeing the patients on a day to day basis, I could utilise the skills that they were telling us…* (ESI1)

## Discussion

### Summary of findings

This study evaluated the feasibility and acceptability of a brief psychological intervention for people with long-term physical health conditions and comorbid subthreshold depression, delivered by trained pharmacy staff within a community pharmacy setting.

Community pharmacies were interested in participating in the study, and supported the training of pharmacy staff to become ESI facilitators. Recruitment of participants to the feasibility study was challenging. Recruitment methods were adapted and new strategies implemented throughout the recruitment period, which was extended beyond the original timeframe [22]. Community pharmacies played an active role in recruitment discussions and offered alternative ways of recruiting people to the study, such as providing study information to those people receiving home-delivered prescriptions. Engagement with the ESI was relatively good with 17 of the 24 (70.8%) recruited participants commencing the ESI. All 17 participants completed a minimum of two sessions, with 10/17 participants completing all six sessions. Retention at 4 months was high (83.3%) and completion of the outcome measures was excellent (100% at baseline, 95 to 100% at the 4-month follow-up). This suggests participants were engaged with the study and were willing to complete study questionnaires.

The study and the ESI made sense to the ESI facilitators, other pharmacy staff and ESI participants. The ESI facilitators valued the ESI training, suggesting that it supported them to develop skills which would help in their routine work within the pharmacy. People with LTCs viewed pharmacy staff as appropriate personnel to talk to about their mood, and to give advice about managing mood, highlighting important personal qualities of the ESI facilitator which made the ESI acceptable. The setting was non-stigmatising and a venue that ESI participants were comfortable with; the fact that the ESI facilitators were often well-known to the ESI participants was a key enabler.

However, there were opportunity costs to the pharmacies which impeded recruitment and impacted on the ability and availability of staff to deliver the ESI to ESI participants. The burden of the recruitment processes impacted on routine work within the pharmacy, and limited the success of the range of study recruitment strategies.

### Comparison with previous literature

Community pharmacies provide people with a link to local health and social care and are a vital part of NHS primary care services [11, 12]. They are accessible and thus well placed to offer opportunistic support to people with a range of health problems including subthreshold depression. In recent decades, the advancement of the pharmacy profession has seen a movement away from a traditional supply function towards more clinically orientated activities [13, 21]. These have been shown to be acceptable developments [32, 33], and a systematic review suggested that patients and the public would find extended services delivered by pharmacies to be acceptable [34]. There is evidence to support the effectiveness of pharmacy-delivered interventions, for example smoking cessation [35, 36]. Whilst no previous studies report pharmacy staff being trained to deliver a psychological intervention to people with mood problems, there is evidence that non-mental health professionals can deliver such psychological interventions [19, 20, 37].

### Strengths and limitations

A strength of this study was the novel-setting, community pharmacies, to deliver a brief psychological intervention to adults with LTCs and subthreshold depression. Community pharmacies were interested in being involved, were engaged with the study and supported training of their staff. The ESI facilitators participated in the ESI training and remained engaged in the study. All reported that the skills learned would be useful in their usual pharmacy work. The qualitative findings provided a valuable learning opportunity and were important in informing the next stage of this research (a pilot RCT).

A limitation of the study is the small sample size which was limited to those of white ethnic origin, although it does reflect the composition of participating community pharmacies in the north east of England. Recruitment was slow, despite the use of a range of recruitment strategies, and additional pharmacies had to be recruited to achieve recruitment figures within the original target [22]. Pharmacy staff identified lack of time and experience of research as barriers to recruiting participants.

### Implications

Learning from the feasibility study contributed to the design of a pilot RCT [22]. This included refinement of recruitment strategies (e.g. increasing opportunities for pharmacy customers to receive the study information, modification and simplification of recruitment materials, and streamlining pharmacy staff recruitment paperwork and training), refinement of the ESI training for ESI facilitators, and modifications of the patient workbook and ESI facilitator support manual.

## Conclusions

This feasibility study informed the design of a pilot RCT [22]. Recruitment within the community pharmacy setting was found to be difficult despite the varied methods of recruitment employed, although qualitative findings helped to identify barriers and enabling factors for participation. Study processes need streamlining to facilitate embedding the study within the community pharmacy setting which in turn would reduce the burden on pharmacies. A variety of recruitment strategies are also required with a need to extend beyond pharmacy-based recruitment approaches and should include wider collaboration with general practitioners (to undertake searches of practice lists) and the wider community and support services. Once recruited, participants engaged well with the ESI and study in general.

Community pharmacies were viewed as an appropriate and non-stigmatising setting in which to deliver preventative brief psychological support to people with LTCs at risk of depression. There were factors within the pharmacies which impeded recruitment, and impacted on the ability and availability of staff to deliver the ESI to ESI participants. The psychological intervention, delivered by trained members of the pharmacy team, was acceptable to people with long-term physical conditions and subthreshold depression.

## Data Availability

Data requests are available in line with the University of York and Tees Esk and Wear Valleys NHS FT standard operating procedures. Requests are to be made via the corresponding author.

## Abbreviations

BA: Behavioural activation
CC: Collaborative care
CHEMIST: Community Pharmacies Mood Intervention Study
ESI: Enhanced Support Intervention
GAD-7: Generalised Anxiety Disorder-7
GP: General Practitioner
LTC: Long-term condition
MINI: Mini International Neuropsychiatric Interview
NHS: National Health Service
PHQ-9: Patient Health Questionnaire-9
PHQ-15: Patient Health Questionnaire-15
RCT: Randomised controlled trial
SD: Standard deviation
SF-12: Short Form- 12
TFA: Theoretical Framework of Acceptability

## References

[1] NHS and Public Health England's Five Year Forward View Plan http://www.england.nhs.uk/ourwork/futurenhs/5yfv-fore/

[2] Mercer SW, Watt GC (2007). The inverse care law: clinical primary care encounters in deprived and affluent areas of Scotland. Ann Fam Med.

[3] World Health Organisation (2008). The global burden of disease: 2004 update.

[4] (NICE). NIfHaCE. Depression: treatment and management of depression in adults. Clinical Guideline 90. London: NICE; [Available from: www.nice.org.uk/guidance/cg90

[5] Cuijpers P, de Graaf R, van Dorsselaer S (2004). Minor depression: risk profiles, functional disability, health care use and risk of developing major depression. J Affect Disord.

[6] Whooley MA, Avins AL, Miranda J, Browner WS (1997). Case-finding instruments for depression: two questions are as good as many. J Gen Intern Med.

[7] Marmott M (2010). The Marmot review final report: fiar society, healthy lives.

[8] McManus S, Meltzer H, Brugha T, Bebbington P, Jenkins R (2009). Adult psychiatric morbidity in England 2007: results of a household survey.

[9] Cuijpers P, Koole SL, van Dijke A, Roca M, Li J, Reynolds CF (2014). Psychotherapy for subclinical depression: meta-analysis. Br J Psychiatry.

[10] Thomson K, Hillier-Brown F, Walton N, Bilaj M, Bambra C, Todd A. The effects of community pharmacy delivered public health interventions on population health and health inequalities: a review of reviews. Prev Med. 2019;124:98–109. 10.1016/j.ypmed.2019.04.003. Epub 2019 Apr 5. PMID: 30959070. 10.1016/j.ypmed.2019.04.003 30959070

[11] Health Do. Implementing the new Community Pharmacy Contractual Framework. London 2005 [Available from: webarchive.nationalarchives.gov.uk/+/www.dh.gov.uk/en/PublicationsandStatistics/Publications/PublicationsPolicyAndGuidance/DH_4109256

[12] Excellence NIfHaC. NICE Guideline Community pharmacies: promoting health and wellbeing. London: NICE; 2018.

[13] Confederation N. Health on the high street: rethinking the role of community pharmacy. Pharmacy and public health task force report Birmingham, UK, NHS Confederation. 2013.

[14] Todd A, Copeland A, Husband A, Kasim A, Bambra C. The positive pharmacy care law: an area-level analysis of the relationship between community pharmacy distribution, urbanity and social deprivation in England. BMJ open. 2014;4:e005764. 10.1136/bmjopen-2014-005764. 10.1136/bmjopen-2014-005764 PMC4156797 25116456

[15] Gilbody S, Lewis H, Adamson J, Atherton K, Bailey D, Birtwistle J (2017). Effect of collaborative care vs usual care on depressive symptoms in older adults with subthreshold depression: the CASPER randomized clinical trial. Jama..

[16] Ekers D, Murphy R, Archer J, Ebenezer C, Kemp D, Gilbody S (2013). Nurse-delivered collaborative care for depression and long-term physical conditions: a systematic review and meta-analysis. J Affect Disord.

[17] Webster LAD ED, Chew-Graham CA. Feasibility of training practice nurses to deliver a psychosocial intervention within a collaborative care framework for people with depression and long term conditions. BMC Nursing 2016;15:17. 10.1186/s12912-016-0190-2 PMC5142437 27980453

[18] Lovell K, Bower P, Gellatly J, Byford S, Bee P, McMillan D (2017). Clinical effectiveness, cost-effectiveness and acceptability of low-intensity interventions in the management of obsessive–compulsive disorder: the Obsessive–Compulsive Treatment Efficacy randomised controlled Trial (OCTET). Health Technol Assess.

[19] Lovell KLJ, Gask L, Bower P, Waheed W, Chew-Graham C (2014). Development and evaluation of culturally sensitive psychosocial interventions for under-served people in primary care. BMC Psychiatry.

[20] Kingstone T, Bartlam B, Burroughs H, Bullock P, Lovell K, Ray M (2019). Can support workers from AgeUK deliver an intervention to support older people with anxiety and depression? A qualitative evaluation. BMC Fam Pract.

[21] Committee PSN. Evaluation of the Healthy Living Pharmacy work programme 2011-2012 2013 [Available from: http://psnc.org.uk/wp-content/uploads/2013/08/HLP-evaluation.pdf.

[22] Littlewood E, Ali S, Badenhorst J, Bailey D, Bambra C, Chew-Graham C (2019). Community Pharmacies Mood Intervention Study (CHEMIST): feasibility and external pilot randomised controlled trial protocol. Pilot Feasibility Stud.

[23] Sheehan DV, Lecrubier Y, Sheehan KH, Amorim P, Janavs J, Weiller E (1998). The Mini-International Neuropsychiatric Interview (MINI): the development and validation of a structured diagnostic psychiatric interview for DSM-IV and ICD-10. J Clin Psychiatry.

[24] Kroenke K, Spitzer RL, Williams JB (2001). The PHQ-9: validity of a brief depression severity measure. J Gen Intern Med.

[25] Kroenke K, Spitzer RL, Williams JB (2002). The PHQ-15: validity of a new measure for evaluating the severity of somatic symptoms. Psychosom Med.

[26] Spitzer RL, Kroenke K, Williams JB, Löwe B (2006). A brief measure for assessing generalized anxiety disorder: the GAD-7. Arch Intern Med.

[27] Ware JE, Kosinski M, Keller SD (1996). A 12-Item Short-Form Health Survey: construction of scales and preliminary tests of reliability and validity. Med Care.

[28] Glaser BG (1965). The constant comparative method of qualitative analysis. Soc Probl.

[29] Ritchie J, Spencer L. Qualitative data analysis for applied policy research. London: Routledge 1994:173–194.

[30] Sekhon M, Cartwright M, Francis JJ (2017). Acceptability of healthcare interventions: an overview of reviews and development of a theoretical framework. BMC Health Serv Res.

[31] Henwood KL, Pidgeon NF (1992). Qualitative research and psychological theorizing. Br J Psychol.

[32] Wiedenmayer K, Summers RS, Mackie CA, Gous AG, Everard M, Tromp D (2006). Developing pharmacy practice: a focus on patient care: handbook.

[33] Edmunds J, Calnan MW (2001). The reprofessionalisation of community pharmacy? An exploration of attitudes to extended roles for community pharmacists amongst pharmacists and General Practioners in the United Kingdom. Soc Sci Med.

[34] Hindi AMK, Schafheutle EI, Jacobs S (2018). Patient and public perspectives of community pharmacies in the United Kingdom: a systematic review. Health Expect.

[35] Thomson K, Hillier-Brown F, Walton N, Bilaj M, Bambra C, Todd A (2019). The effects of community pharmacy-delivered public health interventions on population health and health inequalities: a review of reviews. Prev Med.

[36] Steed L, Kassavou A, Madurasinghe VW, Edwards EA, Todd A, Summerbell CD, et al. Community pharmacy interventions for health promotion: effects on professional practice and health outcomes. Cochrane Database Syst Rev. 2014;CD)112017. 10.1002/14651858.CD011207.pub2 PMC6896091 31808563

[37] Kingstone T, Burroughs H, Bartlam B, Ray M, Proctor J, Shepherd T (2017). Developing a community-based psycho-social intervention with older people and third sector workers for anxiety and depression: a qualitative study. BMC Fam Pract.

